# Effects of a monoclonal antibody against (pro)renin receptor on gliomagenesis

**DOI:** 10.1038/s41598-023-28133-x

**Published:** 2023-01-16

**Authors:** Takeshi Fujimori, Yuki Shibayama, Takahiro Kanda, Kenta Suzuki, Daisuke Ogawa, Ryou Ishikawa, Kyuichi Kadota, Toru Matsunaga, Takashi Tamiya, Akira Nishiyama, Keisuke Miyake

**Affiliations:** 1 Department of Neurological Surgery, Kagawa University Faculty of Medicine, 1750-1 Miki-cho, Kita-gun, Kagawa, 761-0793 Japan; 2 Department of Pharmacology, Kagawa University Faculty of Medicine, Kagawa, Japan; 3 Department of Diagnostic Pathology, Kagawa University Faculty of Medicine, Kagawa, Japan

**Keywords:** Cancer, Molecular biology, Stem cells, Molecular medicine

## Abstract

Glioblastoma is characterized by a strong self-renewal potential and poor differentiated state. We have reported previously that the (pro)renin receptor [(P)RR] is a potential target for glioma therapy by silencing the (P)RR gene. Here, we have examined the effects of a monoclonal antibody against (P)RR on gliomagenesis. Human glioma cell lines (U251MG and U87MG) and a glioma stem cell line (MGG23) were used for the in vitro study. The expressions of the Wnt/β-catenin signaling pathway (Wnt signaling pathway) components and stemness markers were measured by Western blotting. The effects of the (P)RR antibody on cell proliferation, sphere formation, apoptosis and migration were also examined. Subcutaneous xenografts were also examined in nude mice. Treatment with the (P)RR antibody reduced expression of Wnt signaling pathway components and stemness markers. Furthermore, the (P)RR antibody reduced cell proliferation and decreased sphere formation significantly. The treatment also suppressed migration and induced apoptosis. In a subcutaneous xenograft model, systemic administration of the (P)RR antibody reduced tumor volume significantly. These data show that treatment with the (P)RR antibody is a potential therapeutic strategy for treating glioblastoma.

## Introduction

Glioblastoma is an aggressive malignant human brain tumor^1^. The prognosis for patients with glioblastoma is unfavorable. Despite modern treatment protocols such as surgical resection or a combination of radiation therapy and temozolomide chemotherapy, the median survival time is 15 months with only 27% of patients living longer than two years following diagnosis^2,3^. Previous studies have shown that glioblastoma is caused by a small number of specific cancer stem cells (CSCs) that form a tumor, which have potential for high tumorgenicity, self-renewal and multi-differentiation^4–6^. According to the CSCs hypothesis, eliminating glioma stem cells (GSCs) is an attractive therapy for treating glioblastoma^6–8^. Several key transcriptional factors involved in stem cell maintenance are proven to be highly expressed in GSCs. We especially focused on SOX2 (sex determining region Y-box 2), one of the most important stem cell markers^26^.

The Wnt/β-catenin signaling pathway (Wnt signaling pathway) mainly contributes to GSC stemness, proliferation and survival. Since the Wnt signaling pathway is strongly involved in pivotal biological characters of glioblastoma, suppression of the Wnt signaling pathway offers a unique opportunity for attenuating gliomagenesis^9–11^.

The (pro)renin receptor [(P)RR] was discovered and cloned by Nguyen et al.^12^. Previous studies have revealed that (P)RR is an integral component of the Wnt receptor complex and (P)RR binds to Frizzled and LDL receptor- related protein 6 (LRP6) in the Wnt receptor complex involved in the development of the central nervous system^13–15^. Furthermore, Hirose et al. showed that neuron-specific (P)RR knockout mice inhibit stem cell self-renewal^15^. (P)RR is also aberrantly expressed in several cancers including glioblastoma^16–18^. We have shown previously that aberrant (P)RR expression activates the Wnt signaling pathway, which correlates with the malignancy of glioma. In vitro studies have also shown that (P)RR silencing with siRNA reduces the proliferative capacity in several human glioma cells^16^.

More recently, we have developed a monoclonal antibody against the extracellular domain of (P)RR, which interacts with LRP6 of Wnt components^19^. In human pancreatic ductal adenocarcinoma^19^ and colorectal cells^18^, the (P)RR antibody [(P)RR Ab] remarkably suppressed tumorigenesis by inhibiting activation of the Wnt signaling pathway.

In the present study, both in vitro and in vivo studies were conducted to examine the effect of this monoclonal antibody against (P)RR on gliomagenesis. In particular, we have focused on the specific role of (P)RR in the maintenance of glioma stem cells.

## Results

### Positive correlation between SOX2 and (P)RR expression in glioma

**Figure 1:**
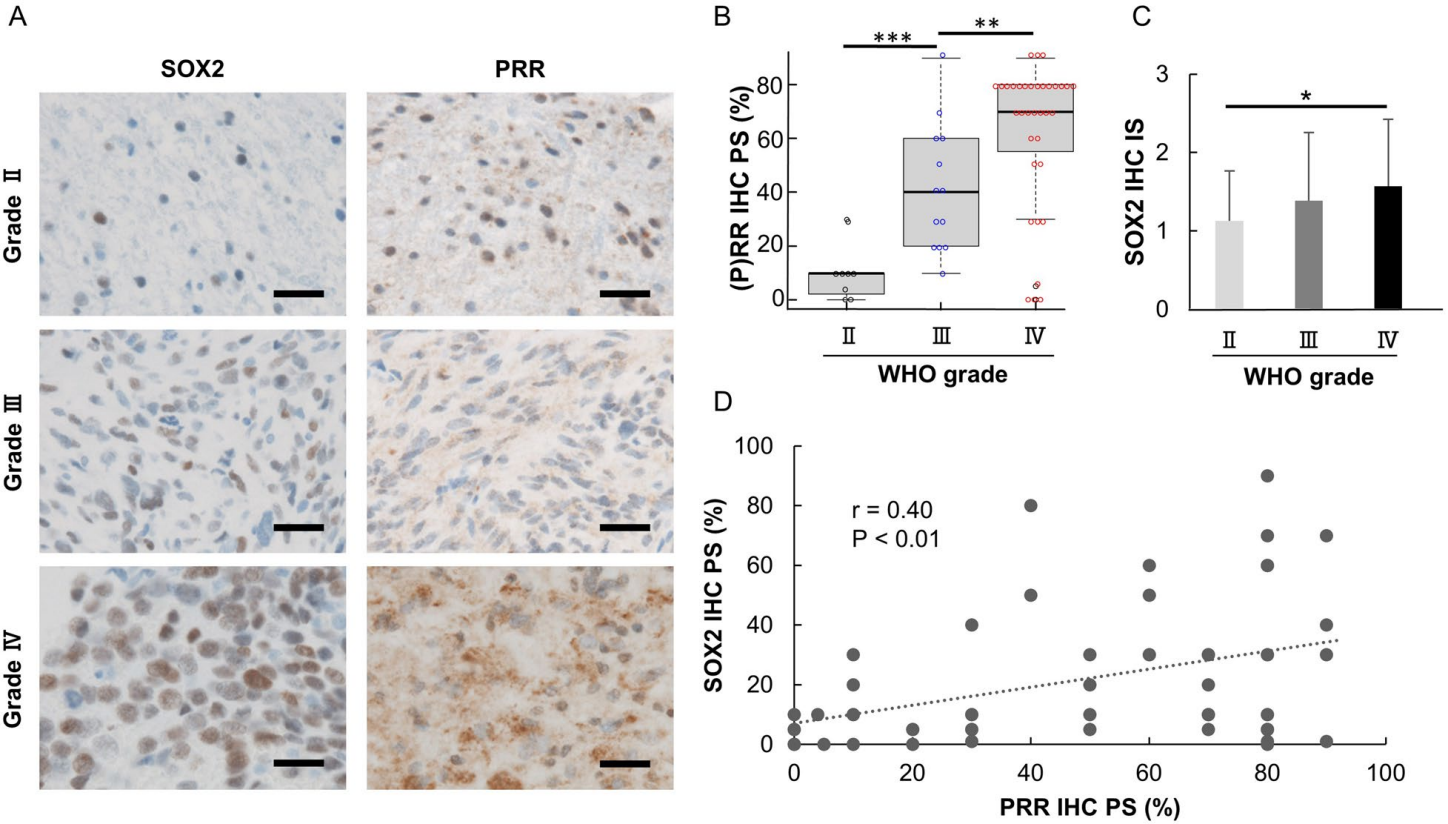
(P)RR and SOX2 expression in human gliomas. (**A**) Expression of SOX2 and (P)RR in representative tumor samples of each WHO grade by immunohistochemistry (scale bar = 50 µm). (**B**) PS of (P)RR and (**C**) IS of SOX2 expression increased according to the WHO grade. (**D**) SOX2 expression weakly correlated with (P)RR expression (*r* = 0.40, *P* < 0.01). *P < 0.05; **P < 0.01; ***P < 0.001.

Immunohistochemistry revealed cytoplasmic (P)RR expression and nuclear SOX2 expression in human glioma tissues (Fig. 1A). Supplementary Table S1 summarizes the detailed characteristics, Proportion score (PS) and intensity score (IS) of the 56 glioma patients. (P)RR expression increased significantly along with the WHO (World health organization) grade (Fig. 1B, supplementary Fig S1B). SOX2 expression tended to increase along with the WHO grade (Fig. 1C, Supplementary Fig S1A). SOX2 expression positively correlated with (P)RR expression (Spearman’s correlation coefficient, *r* = 0.40, *P* < 0.01, Fig. 1D). PS of (P)RR and SOX2 expression increased with tumor malignancy irrespective of isocitrate dehydrogenase (*IDH)*1^R132H^ or 1p19q status (Supplementary Fig. S2). In WHO grade IV, only one case of IDH-mutant type was found, and comparisons in WHO grade IV were omitted.

## Monoclonal (P)RR Ab downregulates the components of the Wnt signaling pathway and stemness markers

**Figure 2:**
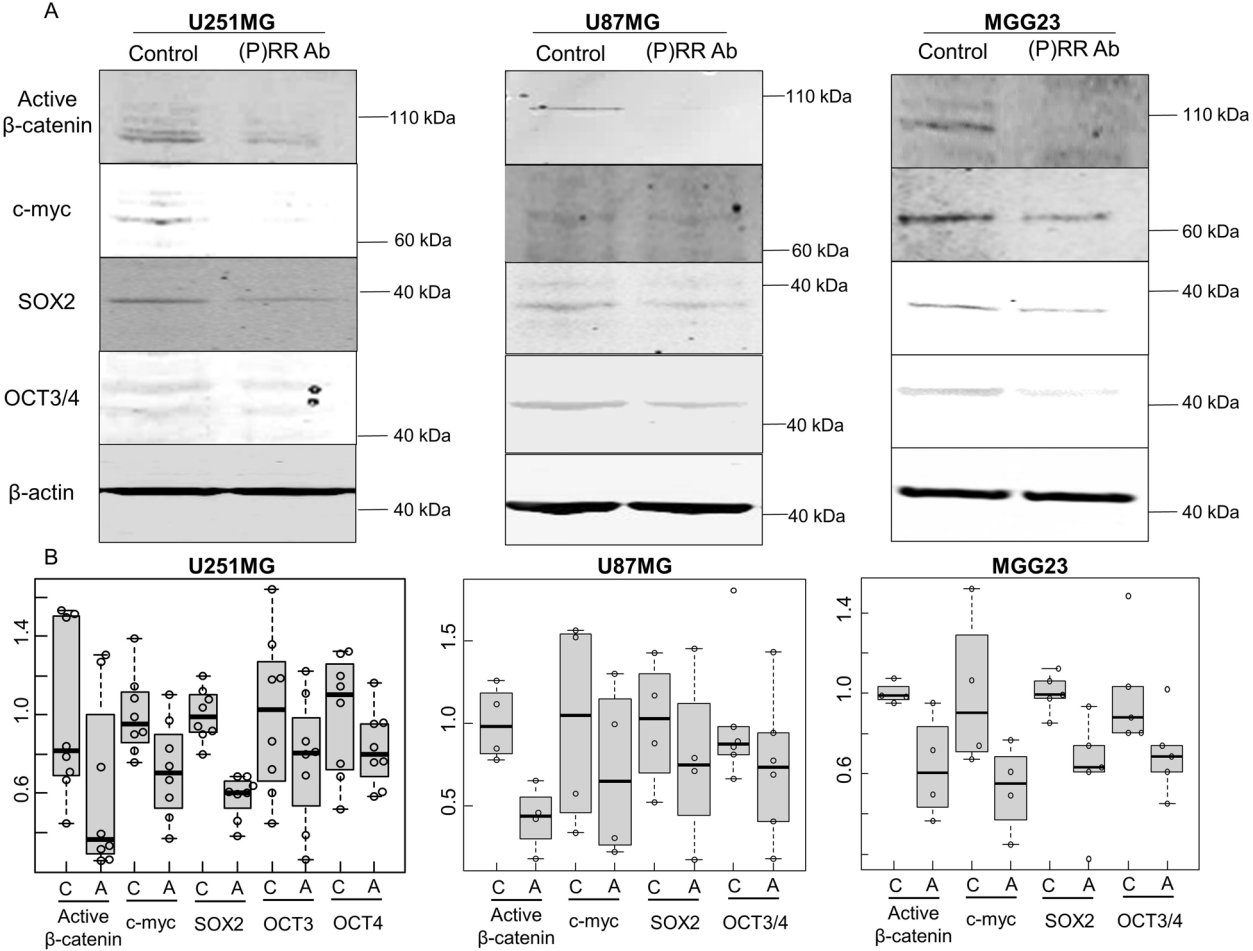
Effect of the (P)RR Ab on Wnt signaling and stemness markers. Using Cells were treated with the (P)RR Ab at 400 µg/mL. (**A**) Representative western blot images showing that treatment with the (P)RR Ab reduced the activity of the Wnt/β-catenin signaling pathway [active β-catenin and c-myc] and expression of stemness markers (SOX2 and Oct3/4) in U251MG, U87MG and MGG23 cells. Original blots are presented in Supplementary Fig. S5. (**B**) Relative protein levels measured using western blotting, quantified and normalized to β-actin levels. *C* control, *A* antibody.

The Wnt/β-catenin signaling pathway is a key regulator of cancer cells^24^. Genes such as cyclin D1 and c-myc are activated following the molecular interaction between β-catenin and lymphoid enhancer binding factor (LEF)/T-cell factor in the nucleus (TCF)^25^. We have already demonstrated that (P)RR is an important component of the canonical Wnt signaling pathway in human glioblastoma cells by using (P)RR siRNA^16^. Therefore, we investigated the effect of a monoclonal antibody against (P)RR on the expression of active (nonphosphorylated) β-catenin and c-myc located downstream of the Wnt signaling pathway. A neutralized activity of (P)RR caused consistent downregulation of β-catenin and c-myc expression in human glioblastoma cell lines U251MG and U87MG and the human GSC cell line MGG23 (Fig. 2A,B, Supplementary Fig. S5). C-myc, Oct3/4 and SOX2 are pivotal transcriptional factors responsible for stem cell maintenance^26^. Expression of these proteins was also downregulated in U251MG, U87MG and MGG23 cells following treatment with the (P)RR Ab (Fig. 2A,B, Supplementary Fig. S5).

### (P)RR Ab suppresses cell viability in human glioblastoma cell lines

**Figure 3:**
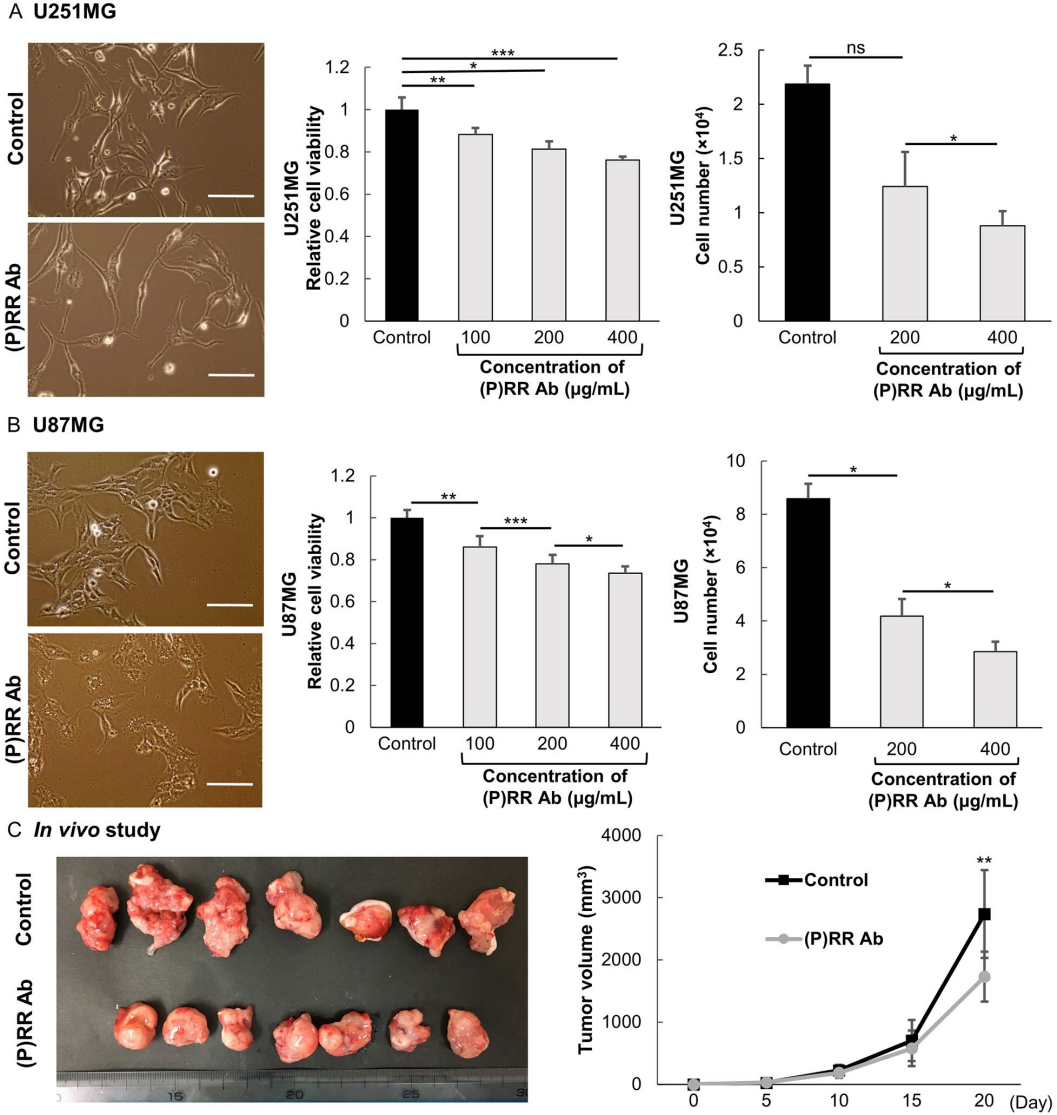
Antiproliferative effects of the (P)RR Ab on human glioblastoma cell lines in vitro and in vivo. The (P)RR Ab dose-dependently reduced cell proliferation in WST-1 (*n* = 3) and cell numbers (*n* = 4) in (**A**) U251MG cells and (**B**) U87MG cells (scale bar = 200 µm). (**C**) In vivo antitumor effect of systemic administration of the (P)RR Ab on engrafted tumors of U87MG cells in athymic nude mice 20 days after injection (left), and time dependent changes in tumor volume (right). The mean tumor volume after 20 days was 1730 ± 399 mL vs. 2739 ± 707 mL (*P* < 0.01, *n* = 7). *P < 0.05; **P < 0.01; ***P < 0.001.

Cell proliferation was evaluated by the water-soluble tetrazolium (WST)-1 assay in U251MG and U87MG cells. This assay revealed that treatment with the (P)RR Ab dose-dependently inhibited the cell viability of U251MG cells [(P)RR Ab treatment of 100 µg/mL (*P* < 0.01), 200 µg/mL (*P* = 0.01) and 400 µg/mL (*P* < 0.001), *n* = , Fig. 3A] and U87MG cells [(P)RR Ab treatment of 100 µg/mL (*P* = 0.04), 200 µg/mL (*P* = 0.03) and 400 µg/mL (*P* = 0.02), *n* = 4, Fig. 3B]. We also investigated proliferation by direct cell counting in human glioblastoma cell lines. The (P)RR Ab decreased significantly the number of viable U251MG cells [(P)RR Ab treatment of 200 µg/mL (*P* = 0.02) and 400 µg/mL (*P* = 0.01), *n* = 3, Fig. 3A] and U87MG cells [(P)RR Ab treatment of 200 µg/mL (*P* = 0.02) and 400 µg/mL (*P* = 0.004), *n* = 3, Fig. 3B]. The (P)RR Ab also affected the cell morphology of human glioblastoma cell lines. U251MG cells treated with the (P)RR Ab had longer dendrites when compared with that of control cells (Fig. 3A). U87MG cells treated with the (P)RR Ab changed cell shape with more edematous than that observed for control cells (Fig. 3B). These findings demonstrated that the (P)RR Ab clearly suppressed human glioblastoma cell viability.

### Antitumor effects of the (P)RR Ab on engrafted U87MG cells

To examine the effects of the (P)RR Ab on glioblastoma tumor growth in vivo, we subcutaneously injected U87MG cells into the right flank of immunodeficient mice and treated the mice with either the (P)RR Ab or supernatant control medium. No treatment-related complications occurred. Twenty days after injection, the mean tumor volume for mice treated with the (P)RR Ab was significantly smaller than the tumors present in the control mice (Fig. 3C). The body weight of mice was not statistically significant between groups (*P* = 0.07, *n* = 7, Supplementary Fig. S3A). Resected tumors treated with control medium had more irregular shapes and a larger necrotic condition than tumors treated with the (P)RR Ab (Fig. 3C). Although there was no significant difference in the mean tumor weight the weights of tumors treated with the (P)RR Ab were slightly reduced (*P* = 0.86, *n* = 7, Supplementary Fig. S3C). These results demonstrated that the (P)RR Ab attenuated the growth of tumors in glioblastoma significantly.

### (P)RR Ab inhibits human glioma stem cell features

**Figure 4:**
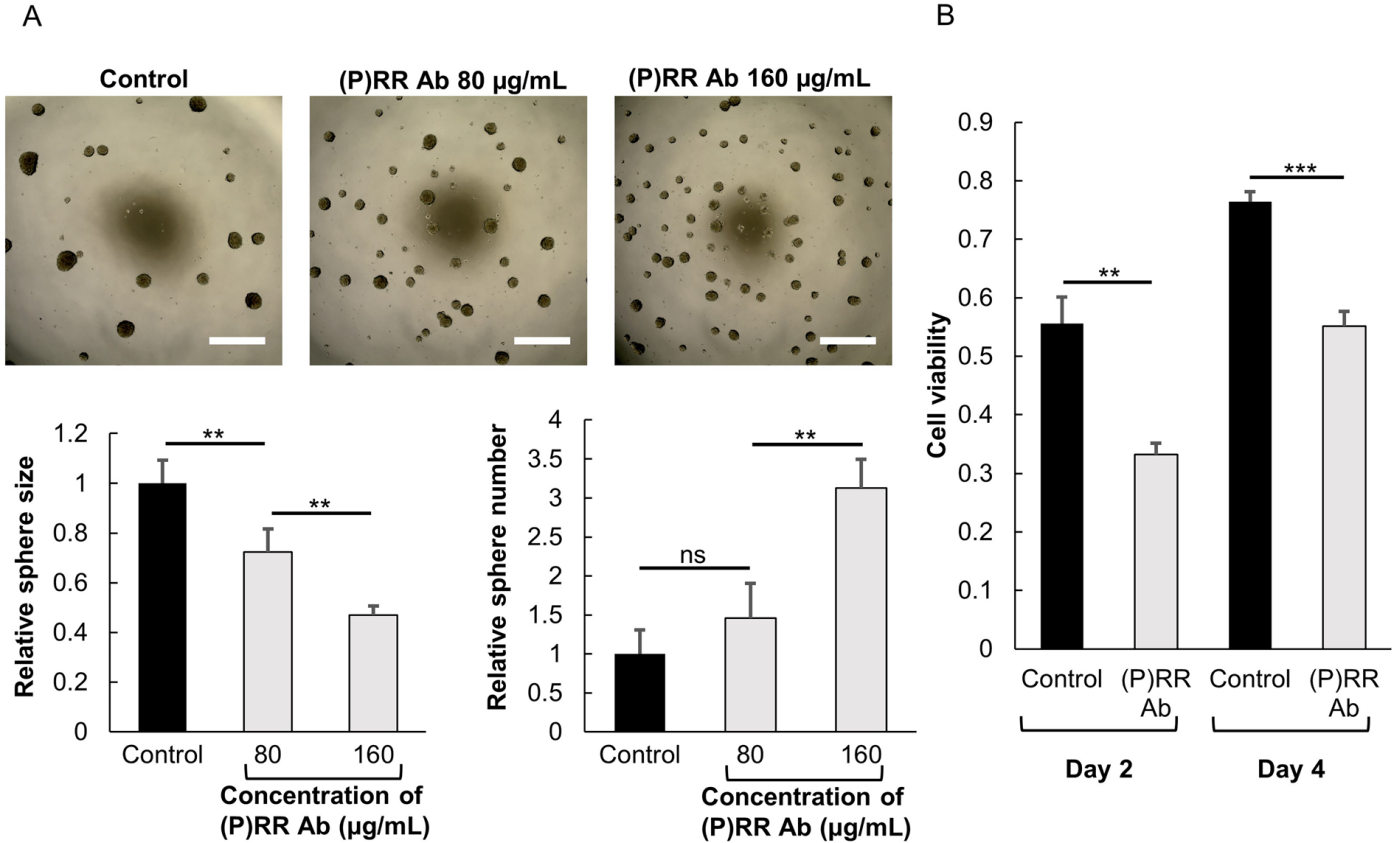
Antiproliferative effects of the (P)RR Ab on the human glioma stem cell line. (**A**) Dose-dependent changes in sphere formation of MGG23 after treatment with the (P)RR Ab (scale bar = 75 µm). The sphere size decreased dose-dependently, while the quantity of the spheres increased (*n* = 4). (**B**) The (P)RR Ab reduced cell proliferation in the MTT assay, both dose-dependently and time-dependently (*n* = 3). *P < 0.05; **P < 0.01; ***P < 0.001.

We performed the sphere formation assay to confirm the effect of the (P)RR Ab. Data showed that the sphere size decreased significantly [(P)RR Ab treatment of 80 µg/mL (*P* = 0.01) and 0 160 µg/mL (*P* = 0.002), *n* = 4, Fig. 4A], and the quantity of spheres increased significantly in MGG23 cells treated with the (P)RR Ab [(P)RR Ab treatment of 80 µg/mL (*P* = 0.08) and 160 µg/mL (*P* = 0.007), *n* = 4, Fig. 4A]. Cell proliferation was evaluated by the MTT [3-(4,5-di-methylthiazol-2-yl)-2,5-diphenyltetrazolium bromide)] assay using MGG23 cells. The (P)RR Ab inhibited cell proliferation in a time-dependent manner [*P* = 0.003 (day 2), and *P* < 0.001 (day 4), *n* = 4, Fig. 4B]. Thus, these data imply that the cell viability of smaller spheres decreases under (P)RR Ab treatment.

### (P)RR Ab induces apoptosis

**Figure 5:**
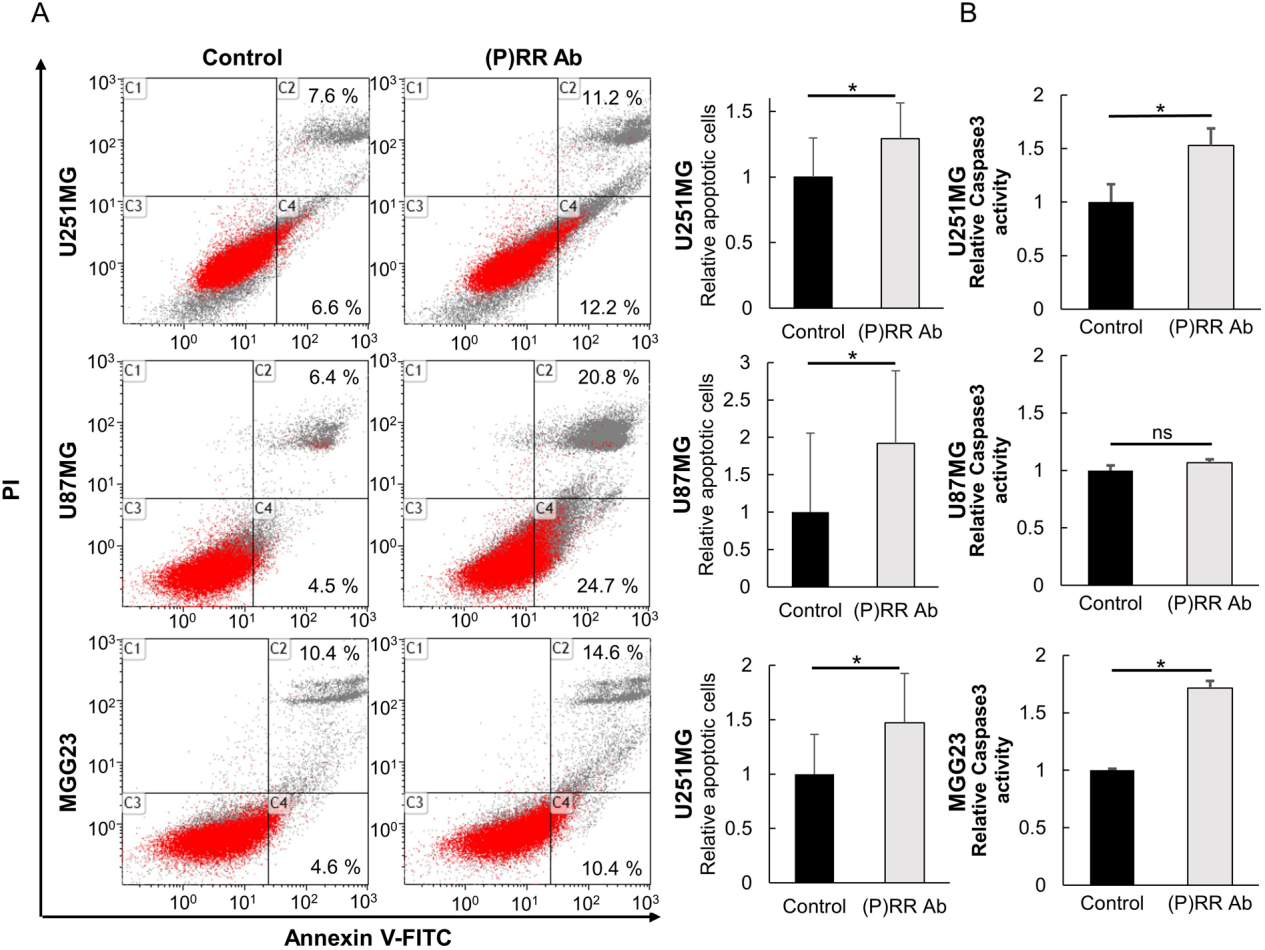
The (P)RR Ab induces apoptosis of glioblastoma cell lines and the glioma stem cell line. (**A**) Detection of early and late apoptosis in U251MG, U87MG and MGG23 treated with control (left) or the (P)RR Ab (right). Cells were stained with FITC-labeled annexin V and PI, and the intensity of fluorescence was assessed at 525 nm (FITC) and 675 nm (PI). C1: dead cells; C2: late-staged apoptosis; C3: viable cells; C4: early-staged apoptosis. A significant increase in the proportion of late-staged apoptotic cells was observed. (**B**) Caspase-3 activity in U251MG, U87MG and MGG23 cells treated with the (P)RR Ab is shown. U251MG and MGG23 cells treated with the (P)RR Ab increased caspase-3 activity significantly (*n* = 3). *P < 0.05.

We performed flow cytometry and caspase-3 assays to evaluate apoptosis in U251MG, U87MG and MGG23 cells. Fluorescein isothiocyanate (FITC)-labeled annexin V was used to identify early apoptotic cells, whereas cells in the late stage of apoptosis were detected by propidium iodide (PI). Thus, viable cells in the early stage of apoptosis were distinguished from late stage apoptotic cells and dead cells by double labeling with annexin V and PI. U251MG, U87MG, and MGG23 cells treated with (P)RR Ab showed a higher percentage of the early- and late-stage apoptotic cells compared with control (U251MG: 10 ± 2.8 and 10.6 ± 5.8% *vs.* 12.9 ± 2.7 and 14.4 ± 8.1%; U87MG: 12.5 ± 13.2 and 13.3 ± 9.1% *vs.* 24 ± 12.1 and 28 ± 12.3%; MGG23: 7.4 ± 2.7 and 11.8 ± 9% *vs.* 10.8 ± 3.3 and 12.4 ± 3.6%) (n = 3, Fig. 5A). Similar data were obtained by the caspase-3 assay. Treatment with the (P)RR Ab significantly increased caspase-3 activity in U251MG cells (*P* = 0.01, *n* = 3) and MGG23 cells (*P* = 0.03, *n* = 3, Fig. 5B). No significant difference was observed in U87MG cells (*P* = 0.1, *n* = 3). These results demonstrated that the (P)RR Ab induced apoptosis in U251MG and MGG23 cells.

### (P)RR Ab suppresses cell migration and adhesion

**Figure 6:**
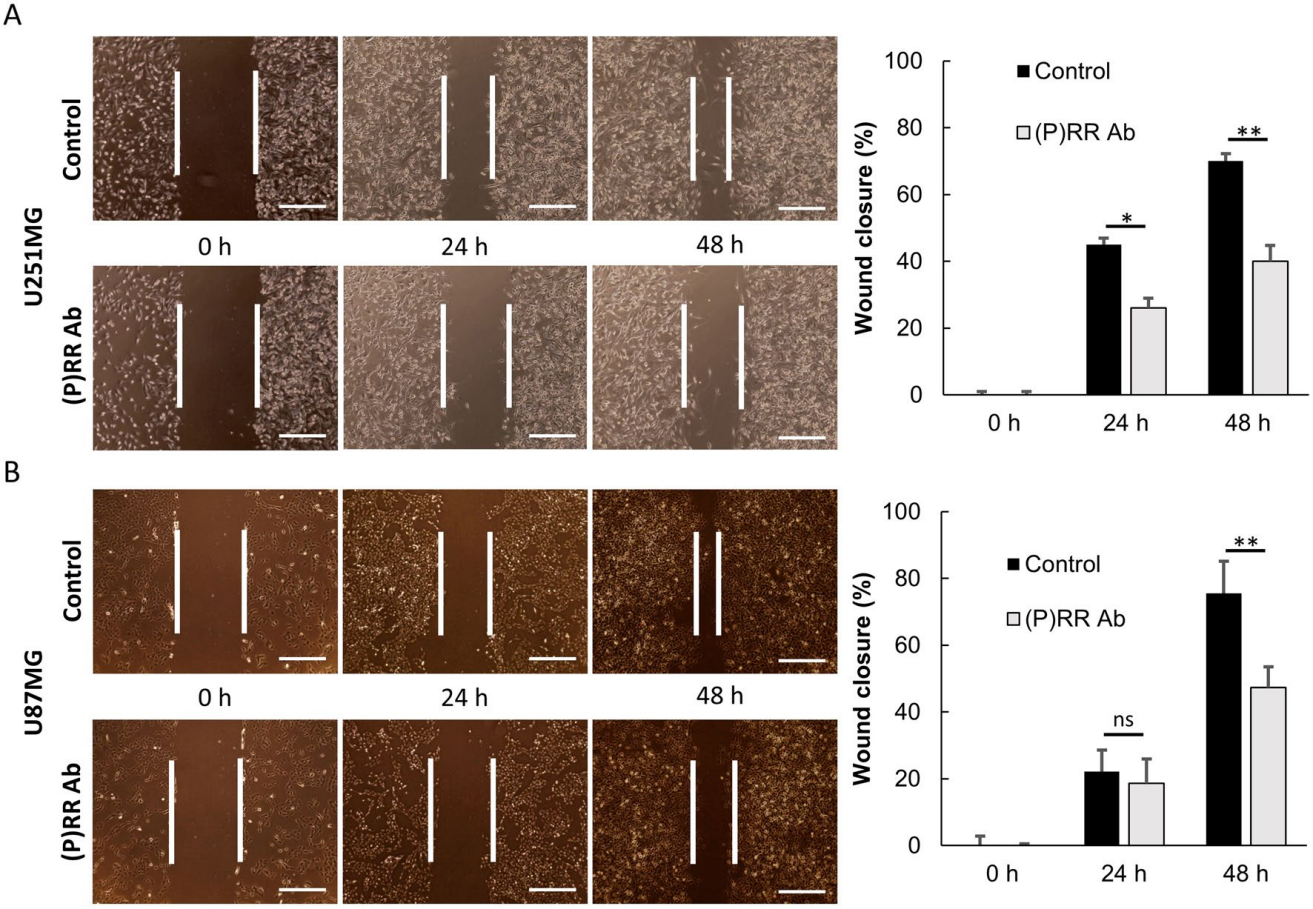
Effects of (P)RR Ab silencing on the migration of glioblastoma cell lines. Migration were evaluated in (**A**) U251MG and (**B**) U87MG by the wound healing assay. Cells were treated with 400 µg/mL of (P)RR Ab. Representative images were captured at 0, 24 and 48 h after scratching (scale bar = 75 µm). The white line shows the borders created by the original scratch. A graphical percentage of the wound closure in (**A**) U251MG and (**B**) U87MG is shown on the right side. The (P)RR Ab decreased cell invasion and migration significantly in U251MG and U87MG (*n* = 5). *P < 0.05; **P < 0.01.

The effect of the (P)RR Ab on cell migration was investigated using the wound healing assay in U251MG and U87MG cells. Compared with that of the control treatment, wound closure in (P)RR Ab-treated cells was attenuated significantly in U251MG cells [24 h after scratching (*P* = 0.02, *n* = 5), and 48 h after scratching (*P* = 0.02, *n* = 5), Fig. 6A] and U87MG cells [24 h after scratching (*P* = 0.6, *n* = 5), and 48 h after scratching (*P* = 0.004, *n* = 5), Fig. 6B].

The effect of the (P)RR Ab in glioma stem-like cells derived from U87MG and U251MG was investigated. Glioma stem-like cells were plated on low-attachment 96-well plates. Cells were cultured in serum-free stem cell medium and then treated with the (P)RR Ab. Four days after treatment, control cells having protrusions were partially attached on the bottom of the plate. In contrast, all cells treated with the (P)RR Ab began to form spheres and float (Supplementary Fig. S4). Attached cells treated with (P)RR Ab were significantly fewer than the control (U251MG, *P* = 0.007, *n* = 4; U87MG, *P* = 0.009, *n* = 4). Thus, the results clearly indicated that the (P)RR Ab suppressed cell adhesion.

**Figure 7:**
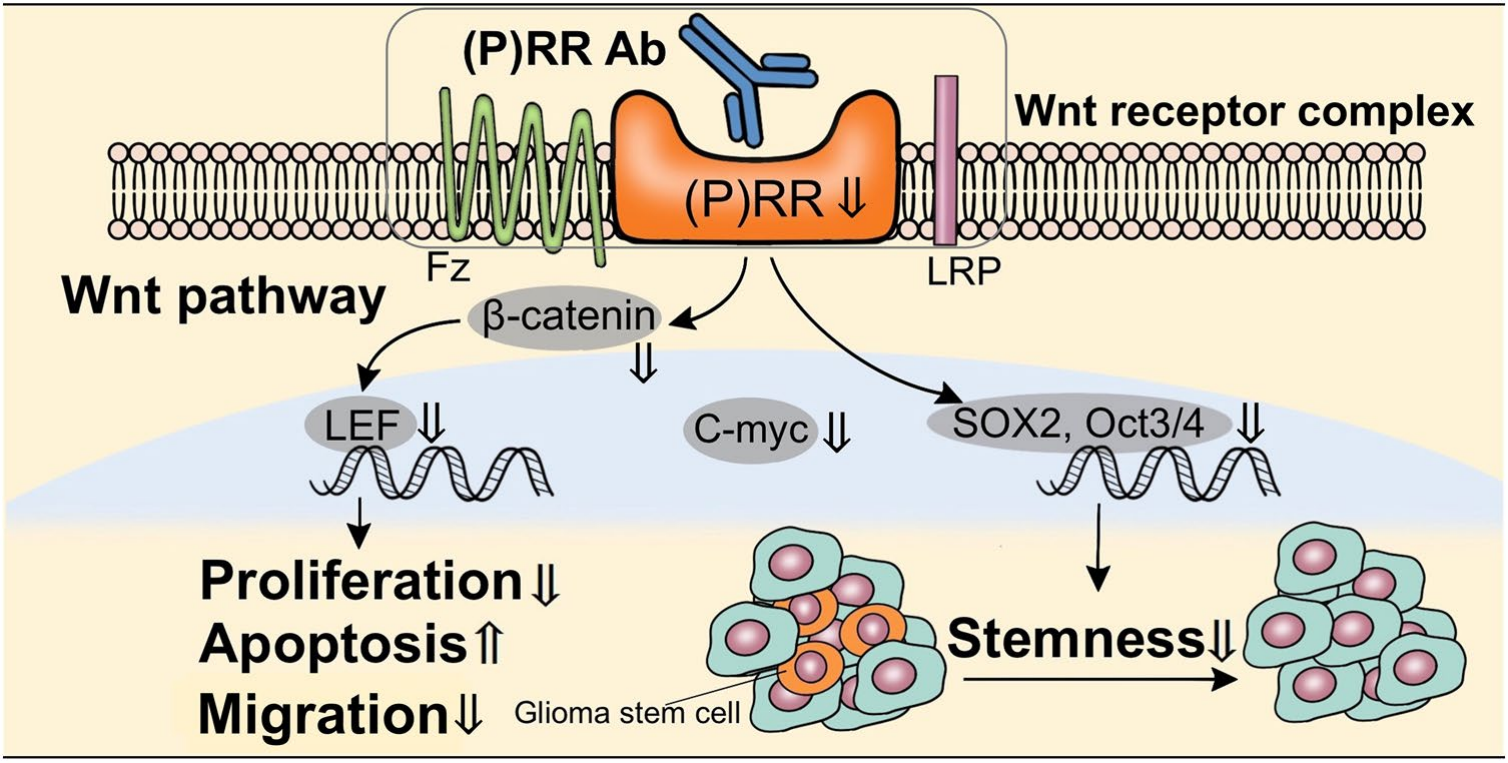
Proposed model for the effect of the (P)RR Ab in glioblastoma. Treatment with the (P)RR Ab decreases cell proliferation and migration and induces apoptosis via inhibition of the Wnt signaling pathway. The (P)RR Ab suppresses SOX2 and Oct3/4 expression and cell proliferation of the glioma stem cell line, which indicates that the (P)RR Ab inhibits stemness of glioma. *Fz* frizzled.

In summary, this study showed that the monoclonal (P)RR Ab suppresses gliomagenesis characterized by cell proliferation, stemness and migration (Fig. 7).

## Discussion

The present study has shown that the monoclonal (P)RR Ab inhibits gliomagenesis by inhibiting the activity of the Wnt signaling pathway. Our data have also indicated the potential contribution of (P)RR to stemness, based on the following observations. First, SOX2 expression positively correlated with (P)RR expression in human glioma tissues. Second, the expression of key molecules that are part of the Wnt signaling pathway and stemness markers were reduced significantly in cells treated with the (P)RR Ab. Third, treatment with the (P)RR Ab inhibited cell proliferation and larger sphere formation. Forth, the (P)RR Ab induced apoptosis and inhibited cell migration and adhesion. Collectively, these data suggest that the (P)RR Ab is a novel promising molecular target drug for treating glioblastoma.

In several cancers including gliomas, activation of the Wnt signaling pathway promotes cell proliferation, migration and invasion, and inhibits apoptosis^29,30^. Cruciat et al.^13^ demonstrated that the extracellular domain of (P)RR interacts with Wnt components LRP6 and Frizzled to activate the Wnt signaling pathway. Our previous studies have indicated that (P)RR plays an important role in the development of glioma by activation of the Wnt signaling pathway^16^. The present study showed that treatment with a monoclonal (P)RR Ab significantly reduced proliferative activity of U251MG, U87MG and MGG23. Our data also showed that administration of the (P)RR Ab reduced the progression of tumor volume significantly in mice xenografted with U87MG cells. There was no significant difference in the mean tumor weight because necrotic tumors were lost when we resected control tumors. Recent studies have shown that a monoclonal (P)RR Ab suppresses cell proliferation by inhibiting the activation of the Wnt signaling pathway in pancreatic adenocarcinoma and colorectal cancer^18,19^. Translocation of β-catenin into the nucleus following aberrant activation of the Wnt signaling pathway enhances cell proliferation through expression of Wnt target genes, such as c-myc and cyclin D1^31,32^. As observed for pancreatic adenocarcinoma and colorectal cancer, we found that treatment with the (P)RR Ab reduced the expression level of c-myc significantly. These data support the hypothesis that the inhibitory effects of the (P)RR Ab on cell proliferation of glioblastoma cells are caused by attenuating the activation of the Wnt signaling pathway. GSCs are thought to be precursors of a variety of malignant gliomas and a small subpopulation of tumor cells that self-renew and proliferate to maintain tumor growth^6,33^. The Wnt signaling pathway has been reported to regulate self-renewal of GSCs, in addition to cell proliferation, migration and differentiation potentially providing an opportunity for therapeutic targeting^34–36^. For example, antihelmintic drug pyrvinum pamoate targeted for Wnt pathway^37^, resveratrol targeted for nuclear β-catenin and c-myc^38^ and tankyrases targeted for Axin^39^ have already been reported as representative inhibitors of Wnt signaling pathway. Furthermore, these therapies also reduce GSC tumorigenicity. (P)RR is located on the upstream of nuclear β-catenin, c-myc and Axin in Wnt signaling pathway, which implies that (P)RR Ab will have comprehensive pharmacological effects of these reagents.

Oct3/4, SOX2 and c-myc are linked to the formation of GSCs, carcinogenesis and tumor progression, which play pivotal roles in the maintenance of GSCs phenotype and stemness^40–44^. In the present study, we especially focused on SOX2 expression, because increased SOX2 levels are associated with a poor outcome for glioma patients^45^. We previously found that (P)RR expression correlates positively with the malignancy of glioma regardless of the presence /absence of *IDH1*^R132H^^16^. Our present data have also revealed that there is a positive relationship between (P)RR and SOX2 expression irrespective of *IDH* or 1p19q status in glioma patients. These data suggest that (P)RR and SOX2 expression is not affected by distinctive mutations of glioma but by grade. In addition, in vitro studies have shown that expression of Oct3/4, SOX2 and c-myc were reduced significantly in U251MG, U87MG and MGG23 cells by treatment with the (P)RR Ab. These data suggest that (P)RR expression affects these molecules responsible for stemness. Our data have also implied that of the MTT assay and the sphere formation assay using MGG23 cells as a human GSC cell line, the (P)RR Ab suppressed cell proliferation and a large sphere formation significantly. In MTT assay, inhibition of proliferation from days 2 to 4 by antibody treatment is not that evident. Thus, the duration of suppression by (P)RR antibody should be considered. Previous studies have indicated that stem cells are characterized by larger spheres, whereas progenitor cells that lost the ability of self-renewal are characterized by smaller spheres^27^, implying that the sphere size is a more important biological feature of stem cells compared to the sphere number. These data have suggested that the sphere size plays a more critical role in the proliferation of sphere-forming cells^28^. Previous studies supported our data. These data indicated that the (P)RR Ab significantly suppressed a large sphere formation and viability of glioblastoma stem cells. These data support the concept that (P)RR regulates stemness in glioma and the (P)RR Ab inhibits biological roles of GSCs.

To further explore the molecular mechanisms by which the (P)RR Ab inhibits glioblastoma cell proliferation, we investigated whether apoptosis is induced by using flow cytometry and caspase-3 assays. Flow cytometry data indicated that treatment with the (P)RR Ab induced apoptosis in these cells. However, in caspase-3 assay using U87MG cells, the effect of (P)RR Ab was limited. Another apoptosis pathway such as NFkB pathway which do not relate to caspase-3 may be involved in the effects of (P)RR Ab in U87MG cells^46^. Data indicated that treatment with the (P)RR Ab induced apoptosis in these cells. Recent studies have indicated a possible relationship between glioblastoma migration and the GSC subpopulation^47^. The activity of the Wnt signaling pathway may also contribute to migratory in many malignancies^34^. In our wound healing migration assay, treatment with the (P)RR Ab suppressed cell migration of U251MG and U87MG. Furthermore, treatment with the (P)RR Ab suppressed cell adhesion of U251MG and U87MG cultured under stem cell conditions. Taken together, these data suggest that the (P)RR Ab suppress glioblastoma through multiple mechanisms.

There are several limitations in the present study. The main limitation is the small sample size of patients and single institute retrospective design in immunohistochemistry (IHC) study. Second, the only one cell line was used in *vivo* study and further study using orthotopic intracranial models are required to examine whether therapeutic (P)RR antibodies pass through blood brain barrier. Third, safety of (P)RR Ab administration needs to be confirmed for clinical application. Future studies will be performed to overcome these limitations.

In conclusion, the present study has revealed that a monoclonal (P)RR Ab inhibited gliomagenesis such as cell proliferation, stemness and migration. These data suggest that (P)RR is a novel therapeutic target via inhibition of stemness and the Wnt signaling pathway.

## Materials and methods

### Glioma primary tissues

Glioma tumor specimens were obtained from patients who underwent a craniotomy at Kagawa University Faculty of Medicine between 2013 and 2019. Patients that underwent chemotherapy or radiotherapy before surgery were excluded from the study. Fifty-six patients were included for IHC analysis.

This study was conducted in accordance with the principles of the Declaration of Helsinki and approved by the institutional review board of Kagawa University Hospital (approval number 2020-118). The institutional review board of Kagawa University Hospital approved that the requirement for informed consent was waived in this study because patient identifiers were completely removed and data were collected retrospectively.

### IHC for (P)RR and SOX2

Representative sections were prepared from the resected glioma samples. Tissues were fixed in 10% buffered formalin for 24–48 h and embedded in paraffin. Immunohistochemical staining was performed in an automated system using the Ventana BenchMark ULTRA (Roche Diagnostics, Basel, catalog #518-108496), according to the manufacturer’s instructions. For the primary antibody, we used the anti-human (P)RR rabbit polyclonal antibody^20,21^ with a 1:6000 dilution, and the anti-SOX2 (SP76) rabbit monoclonal antibody (Cell Marque, Rocklin, CA, #371R-1) with a 1:100 dilution. PS and IS were blindly scored by two pathologists. PS was counted using the hotspot method, i.e., percentage areas of positive staining were calculated as the average of the three most strongly stained areas of one section. IS was based on staining intensity: 1 = weak, 2 = moderate and 3 = strong.

### Cell culture

Human glioblastoma cell lines (U251MG, U87MG) were obtained from the American Type Culture Collection. These cell lines were incubated in Dulbecco’s Modified Eagle’s Medium (Thermo Fisher Scientific, Waltham, MA, catalog #11965118) with 10% fetal bovine serum (Sigma-Aldrich, St. Louis, MO, catalog #7524-500ML) at 37 °C under 5% CO_2_/95% air in a humidified incubator. The human glioblastoma derived cancer stem cell line, MGG23 cells, were provided by Dr. Hiroaki Wakimoto at Massachusetts General Hospital^22,23^. MGG23 cells were cultured in neurobasal medium (Thermo Fisher Scientific, catalog #21103049) supplemented with L-glutamine (3 mM; Thermo Fisher Scientific, catalog #A2916801), B27 supplement (Thermo Fisher Scientific, catalog #17504044), EGF (20 ng/mL; R&D Systems, Minneapolis, MO, catalog #236-EG-200) and FGF (20 ng/mL; Peprotech, Cranbury, NJ, catalog #100-41) to establish neurosphere cultures enriched for GSCs.

Glioma stem-like cells were cultured and isolated from U251MG and U87MG glioma cell lines by using serum-free medium, as described above.

### Generation of the (P)RR Ab

We used the monoclonal (P)RR Ab in both in vitro and in vivo studies. As shown previously, the (P)RR Ab was designed to target the extracellular domain of the (P)RR by using the rat lymph node method^19^. Control supernatant medium was used as the vehicle treatment.

### Western blotting

Protein concentrations were measured using the Bradford method. Total protein extracts (30 µg) were electrophoretically separated on 10% SDS–polyacrylamide gels and transferred onto nitrocellulose membranes. Blots were blocked with blocking solution (LI-COR, Lincoln, NE, catalog #927-40000). The primary antibodies used (1:1000 dilution in blocking solution) were the anti-active-β-catenin (Millipore, Billerica, MA, catalog #05-665), c-myc (Santa Cruz Biotechnology, Dallas, TX, catalog #sc-40), SOX2 (Cell Signaling, Danvers, MA catalog #2748) and Oct (octamer-binding transcription factor) 3/4 (Santa Cruz Biotechnology, catalog #sc-5279). After incubation with secondary antibodies (1:1000 dilution in blocking solution) coupled with infrared dyes (IRDye 800 goat anti-rabbit immunoglobulin G [IgG], IRDye 680 goat anti-mouse IgG and IRDye 680 donkey anti-goat IgG), protein expression was detected using an Odyssey scanner (LI-COR, catalog #9141-00). The membranes were reprobed with an antibody for β-actin (Sigma-Aldrich, catalog #A5441) to confirm equal protein loading. Protein bands were quantified using ImageJ software (National Institutes of Health).

### Cell proliferation assay

WST-1 assay was performed to determine the cell proliferation of U251MG and U87MG cell lines according to the manufacturer’s instructions (Takara Bio, Shiga, catalog #MK400). First, 8 × 10^3^ cells were seeded onto the wells of 24-well plates. After incubation for 72 h, treatment with the (P)RR Ab at 100, 200 and 400 µg/mL was performed. After a further 72 h, 50 µL of the WST-1 reagent was added to the 500 µL cell culture medium in each well and incubated for 1.5 h at 37 °C. Finally, the absorbance was measured at 450 nm by using a microplate reader (Corona Electric Co., Ltd, Ibaraki, catalog #SH-9000).

The MTT assay was performed using the MGG23 cell line. 1.5 × 10^4^ cells per well were plated in 96-well plates and treated with the (P)RR Ab at 200 µg/mL. After 48 h, the MTT solution was added to each well and cells were incubated for 2 h at 37 °C. Thereafter, formazan was solubilized in absolute ethanol, and the absorbance was measured at 595 nm by using a microplate reader (Corona Electric Co., Ltd, Ibaraki, catalog #SH-9000).

### Direct cell counting

Direct cell counting was performed using U251MG and U87MG. 8 × 10^4^ cells were seeded onto the wells of six-well plates. After incubation for 48 h, treatment with the (P)RR Ab at 100, 200 and 400 µg/mL was performed. After a further 48 h incubation, direct cell counting was performed to determine cell numbers. Then, the cells were treated with 0.25% trypsin–EDTA (Thermo Fisher Scientific, catalog #R001100), centrifuged and the cells resuspended. The cell suspensions were stained with AccuStain (NanoEnTek, Seoul, catalog #ADR-1000). Aliquots of the cell suspensions (20 µL) were then loaded into an AccuChip 4× Counting kit (NanoEnTek, catalog #AD4K-200) and viable cells (per mL) were counted using an automated cell counter (NanoEnTek, catalog #ADAM-MC).

### In vivo tumorigenicity studies

Experimental protocols and animal care were performed according to the guidelines for the care and use of animals established by Kagawa University, and the principles of the Declaration of Helsinki. The animal Experimentation Ethics Committee at Kagawa University approved the experimental protocols (approval number: 2020-18648-2). Five week old male BALB/c (nu/nu) nude mice were purchased from CREA (Tokyo, Japan) and were maintained in specific pathogen free animal facilities under a controlled temperature (24 ± 2 °C) and humidity (55 ± 5%) with a 12-h light–dark cycle. Mice were bred by standard chow and water ad libitum. U87MG cells were grown in 15 cm^2^ dishes, trypsinized and centrifuged to collect cell pellets after reaching a confluency of 70–80%. The pellets were diluted with PBS (phosphate-buffered saline) to a final concentration of 1 × 10^6^ cells/200 µL. Cells were inoculated by subcutaneous injection into the right flank of mice previously anesthetized via intraperitoneal administration of ketamine (100 mg/kg)/xylazine (10 mg/kg). Mice were treated with 200 µg/mL of (P)RR Ab diluted in PBS via intraperitoneal injection every 3 days over a 20-day period from the 3rd day after cell transplantation. Control supernatant medium was served as the vehicle treatment. Tumor growth was monitored by measuring the diameter of the local tumors at the implant site with a digital caliper every 5 days up to the end of the experiment. Tumor volumes were calculated according the formula: length × width^2^ × 0.5.

### Sphere formation assay

GSC spheres were enzymatically dissociated to single cells and placed in 96-well plates at an optimal density (1 × 10^4^ cells) in nonadherent conditions and treated with the (P)RR Ab at 200 µg/mL. After 48 h, cells were imaged using an EVOS XL Core microscope (Thermo Fisher Scientific, catalog #12562751). ImageJ (National Institutes of Health) was used to analyze the size and quantity of spheres.

### Detection of apoptosis using annexin V and propidium iodide

After treatment with the (P)RR Ab, U251MG and U87MG cells were incubated for 96 h, whereas MGG23 cells were incubated for 48 h. U251MG and U87MG cells were then treated with 0.25% trypsin–EDTA, centrifuged, washed twice in PBS and resuspended in annexin-binding buffer (1×) according to the manufacture’s protocol (Nacalai tesque, Kyoto, catalog #15342-54). MGG23 cells were treated with TrypLE Express (Thermo Fisher Scientific, catalog #12604013). Next, the cells were stained with FITC-labeled annexin V and PI working solution for 15 min at room temperature, after which 400 µL of annexin-binding buffer was added. Flow cytometry was performed and emissions at 525 nm (FITC) and 675 nm (PI) were monitored.

### Detection of apoptosis by the caspase-3 assay

Forty-eight hours after treatment with the (P)RR Ab, caspase-3 activity was detected according to the manufacture’s protocol (Medical & Biological Laboratories, Nagoya, catalog #4800). Reaction buffer (2×) and the caspase-3 substrate were added to cell lysates, which were injected into the wells of 96-well plates and incubated at 37 °C for 8 h. The absorbance was then measured at 405 nm with a microplate reader (Corona Electric Co., Ltd, Ibaraki, catalog #SH-9000).

### Wound healing assay

U251MG and U87MG cells were grown to confluence in 12-well plates to evaluate cell migration by the wound healing assay. A thin “wound” was introduced by scratching the monolayer with a sterile 200 µL pipette tip. Wells were washed with PBS to remove dead cells and debris, and the medium was replaced with serum-free medium for culturing. Cells were treated with the (P)RR Ab. Migration of cells into the gap was imaged over hours. The wound gap was measured and expressed as a percentage of wound closure. Five gap distances were randomly measured using ImageJ software.

### Statistical analysis

Statistical analyses were performed with EZR software (Saitama Medical Center, Jichi Medical University, Saitama, Japan), which is a graphical user interface for R (The R Foundation for Statistical Computing, Vienna, Austria). Univariate analysis was conducted using the Mann–Whitney U test or unpaired t-test for parametric data. Spearman’s correlation coefficient was used to assess association between variables. *P* < 0.05 was considered significant.

## Supplementary Information

Supplementary Information.

## Data Availability

On reasonable request, derived data supporting the findings of this study are available from the corresponding author.
