# Supplementary material for: Effects of a monoclonal antibody against (pro)renin receptor on gliomagenesis
Source: Sci Rep. 2023 Jan 16;13:808. doi: 10.1038/s41598-023-28133-x (PMC9842725; doi:10.1038/s41598-023-28133-x)
Supplement: Supplementary file 1 — Supplementary Information. [file 41598_2023_28133_MOESM1_ESM.pdf]

Title: Effects of a monoclonal antibody against (pro)renin receptor on gliomagenesis

Authors: Takeshi Fujimori, Yuki Shibayama, Takahiro Kanda, Kenta Suzuki, Daisuke Ogawa, Ryou Ishikawa, Kyuichi Kadota, Toru Matsunaga, Takashi Tamiya , Akira Nishiyama, and Keisuke Miyake

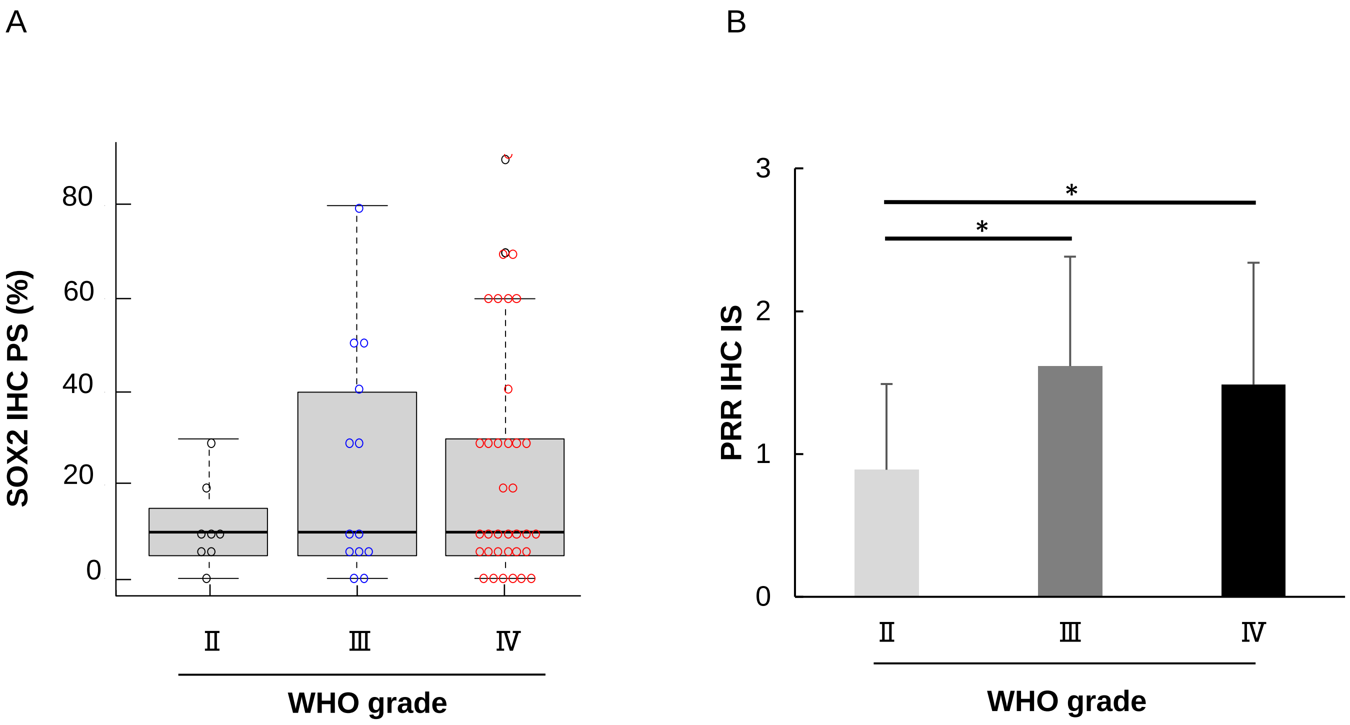

Supplementary Fig. S1. SOX2 and (P)RR expression in human gliomas. (A) PS of SOX2 and (B) IS of (P)RR expression increased with tumor malignancy. *: $P < 0.05$.

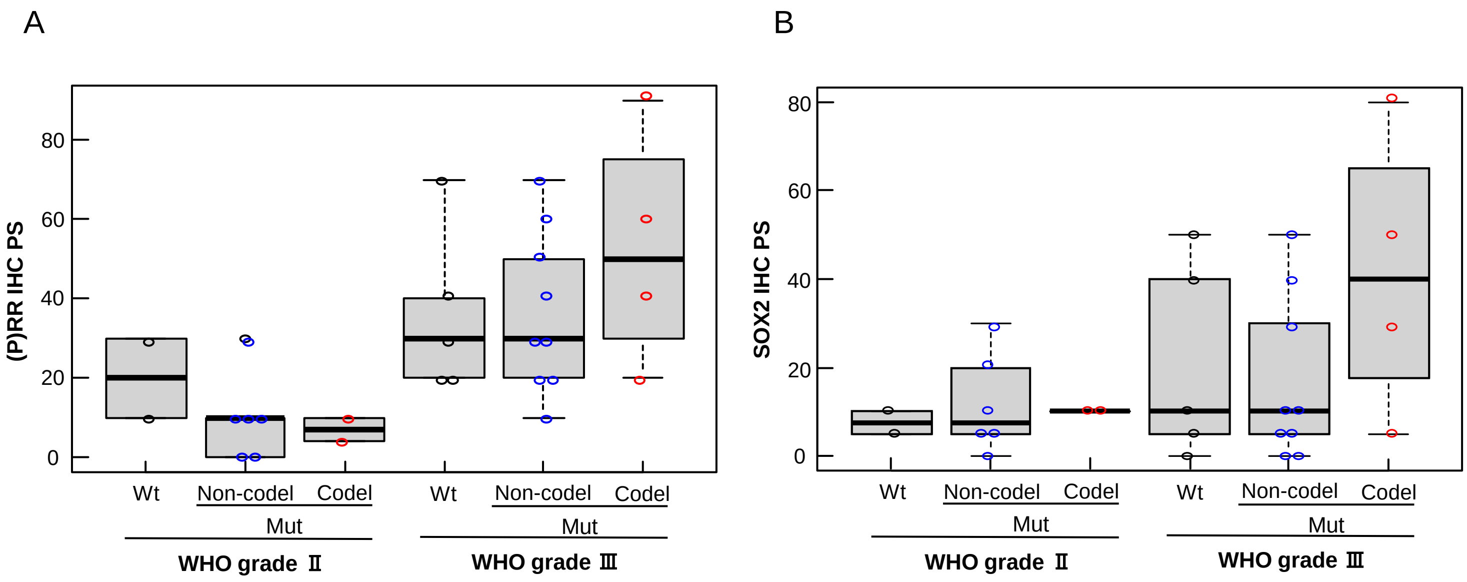

Supplementary Fig. S2. The relationship between (P)RR and SOX2 expression and presence of each *IDH* and 1p19q status. (A) PS of (P)RR expression increased with tumor malignancy irrespective of *IDH* or 1p19q status [the mean PS was 20 ± 14.1% for Wt, 10 ± 11 for Non-codel and 7 ± 4.2 for Codel (grade II) and 36 ± 20.7% for Wt, 36.7 ± 20 for Non-codel and 52.5 ± 29.9 for Codel (grade II)] (B) PS of SOX2 expression increased with tumor malignancy irrespective of *IDH* or 1p19q status [the mean PS was 7.5 ± 3.5% for Wt, 11.7 ± 11.2 for Non-codel and 10 ± 0 for Codel (grade II) and 21.0 ± 22.5% for Wt, 16.7 ± 18.5 for Non-codel and 41.3 ± 31.7 for Codel (grade II)]. Codel, 1p19q codeletion; *IDH*, isocitrate dehydrogenase; IHC, immunohistochemistry; IS, intensity score; Non-codel, non-1p19q codeletion; Mut, *IDH*-mutant type; (P)RR, (pro)renin receptor; PS, proportion score; WHO, World Health Organization; Wt, *IDH*-wild type.

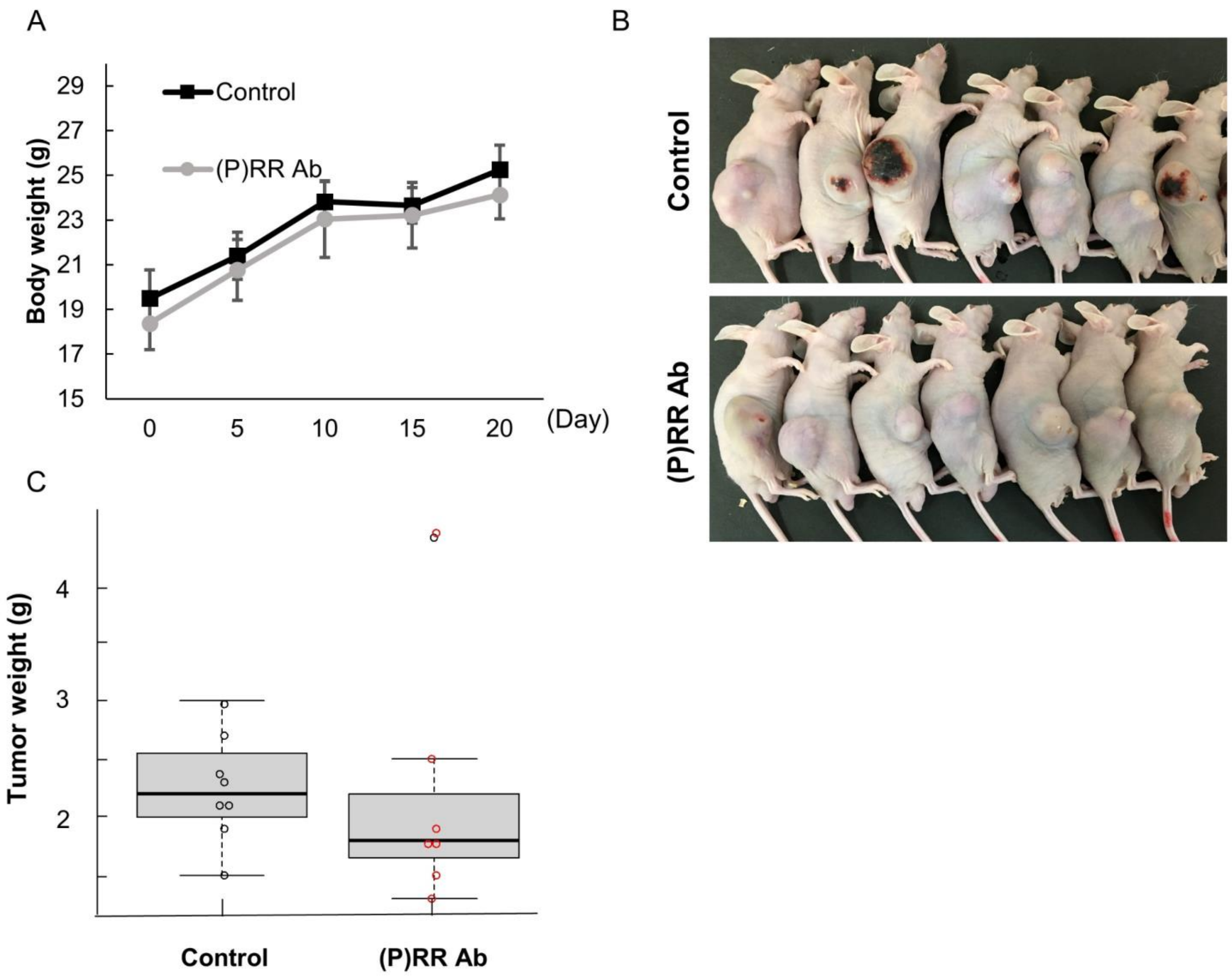

Supplementary Fig. S3. The (P)RR Ab inhibits xenograft tumor growth potential in a nude mouse model. (A) Time dependent changes of the mean body weight. There was no significance between the groups 20 days after injection (24.1 ± 1.1 g *vs*. 25.3 ± 1.1 g, $P = 0.07$, $n = 7$). (B) Representative picture of the engrafted tumor just before sacrifice. (C) Xenograft tumor weight after sacrifice. Tumor weight treated with the (P)RR Ab was mildly suppressed (2.25 ± 0.47 g *vs*. 2.17 ± 1.05 g, $P = 0.86$, $n = 7$).

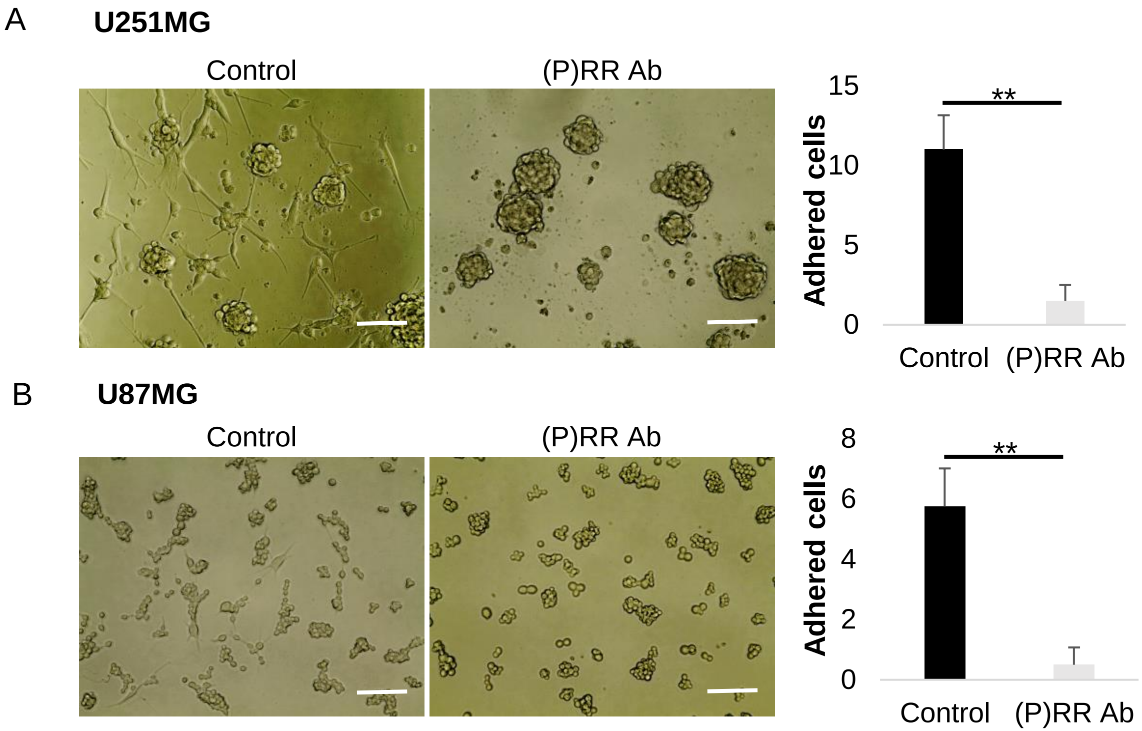

Supplementary Fig. S4. The (P)RR Ab inhibits cell adhesion in glioma stem-like cells. Glioma stem-like cells produced from (A) U251MG and (B) U87MG were treated with the (P)RR Ab at 400µg/mL. Representative pictures were captured four days after treatment (scale bar = 200 µm). Treatment with the (P)RR Ab suppressed cell adhesion. **: $P < 0.01$.

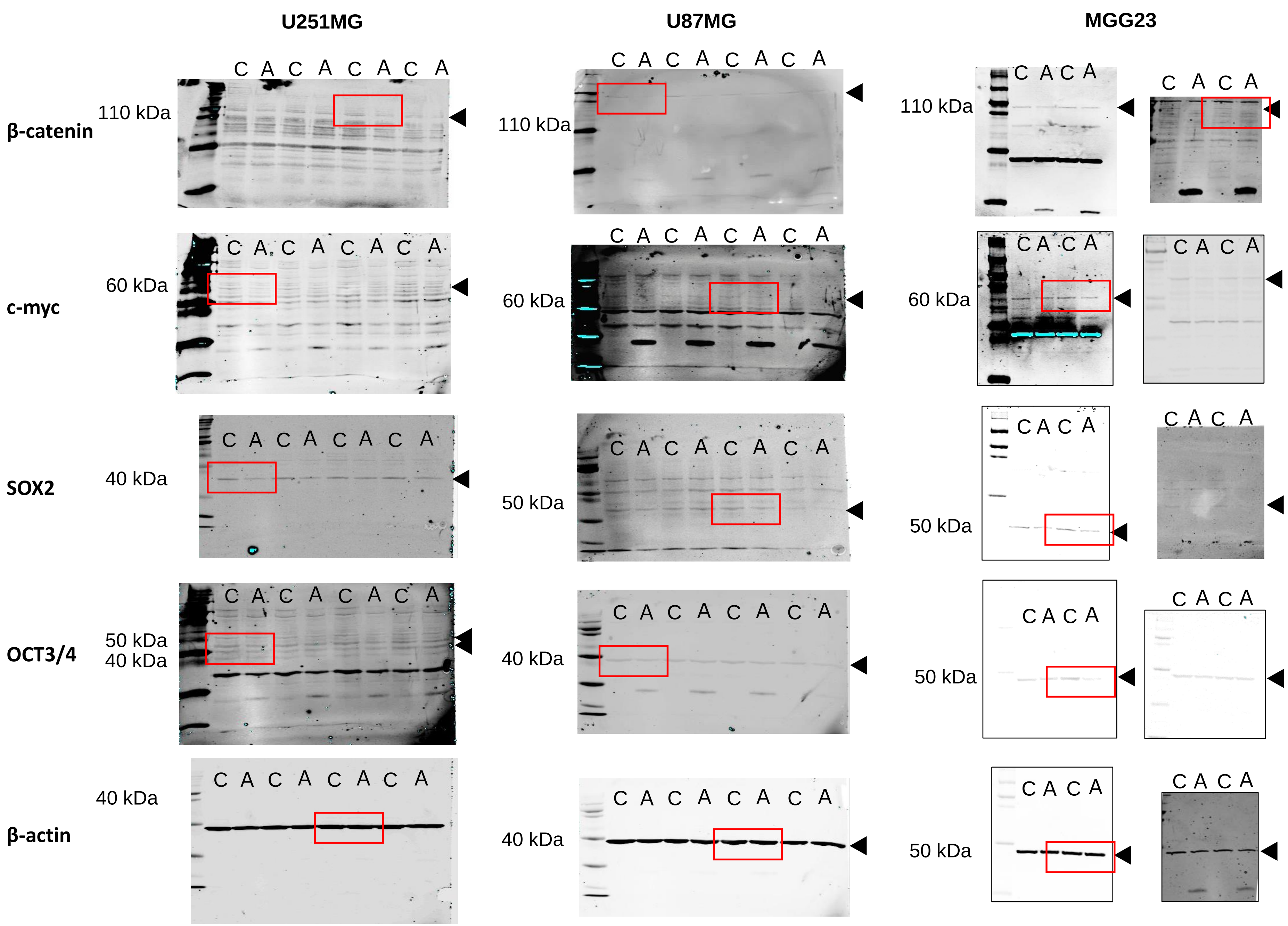

Supplementary Fig. S5. Western blot imaged in U251MG, U87MG, MGG23

The area of the original blots in the Fig. 2 is framed in red. C: Control, A: (P)RR antibody

Supplementary Table S1. Basic characteristics and results of immunohistochemistry in 56 glioma patients

WHO grade	IDH or 1p19q status	No. of patients	Age	SOX2 IHC PS	SOX2 IHC IS	(P)RR IHC PS	(P)RR IHC IS
Ⅱ	Total	8	46	11	1.1	9	0.9
	Wt	2	63	8	1.5	20	1.5
	Non-codel	6	46	12	1	10	0.8
	Codel	2	44	10	1.5	7	1
Ⅲ	Total	13	50	24	1.4	42	1.6
	IDH wild	5	60	21	1.2	36	2
	Non-codel	9	55	17	1.1	37	1.9
	Codel	4	43	41	1	53	1
Ⅳ	Total	35	69	24	1.6	63	1.5
	Wt	34	69	23	1.5	63	1.5
	Mut	1	39	60	3	60	1

Codel, 1p19q codeletion; IDH, isocitrate dehydrogenase; IHC, immunohistochemistry; IS, intensity score; Non-codel, non-1p19q codeletion; Mut, IDH-mutant type; (P)RR, (pro)renin receptor; PS, proportion score; WHO, World Health Organization; Wt, IDH-wild type.
